# Epispadias associated with urethral duplication: Our practice

**DOI:** 10.1016/j.ijscr.2020.05.002

**Published:** 2020-05-27

**Authors:** Ramiz Polukhov, Vugar Mahammadov, Madina Baghirli

**Affiliations:** Department of Pediatric Surgery, Azerbaijan Medical University, Baku, Azerbaijan

**Keywords:** Urethral duplication, Epispadias, Congenital urethral anomalies

## Abstract

**Introduction:**

Urethral duplication and epispadias are one of the rare anomalies of the genitourinary system. Sometimes, urethral duplication can also be associated with anorectal malformations, epispadias, hypospadias, bladder doubling, bladder exstrophy and other urinary excretory system anomalies.

**Presentation of case:**

In this article we report three years old male patient with incomplete urethral duplication and epispadias. During our examination there was detected the second - accessory urethra along with the main urethra on the dorsal surface of the penile. The accessory urethra was stripped from the surrounding tissues to the root of the penile and it became apparent that the accessory urethra was merged into the main urethra at the same level. The accessory urethra was closed by suturing and removed from that part. Then, by using 7.0 pds thread there was performed urethroplasty.

**Discussions:**

Urethral duplication is one of the rarely found congenital anomalies in the genitals. Duplications in the sagittal plane can be complete or incomplete, dorsal or ventral, and may be associated with other congenital urogenital anomalies. An epispadias is a rare type of malformation of the penis in which the urethra ends in an opening on the upper aspect of the penis. Despite the fact that there are many considerations about their embryology, the reason of appearance of these anomalies is still unknown.

**Conclusions:**

While planning of surgical treatment of such common anomalies along with the good cosmetic results the lower urinary excretory system functions should be considered as well.

## Highlights

• Urethral duplication is one of the rarely found congenital anomalies in the genitals.
• An epispadias is a malformation of the penis in which the urethra ends in an opening on the upper aspect of the penis.
• In this article we have reported three years old male patient with incomplete urethral duplication and epispadias.

## 1 Introduction

Urethral duplication is a very rare congenital anomaly of genital organs that occurs more frequently in males than females. It is divided into complete and incomplete forms. Despite the fact that there are many considerations about the embryology of this anomaly, the etiopathogenesis of its various forms is still unclear [[1], [2], [3]]. Sometimes, urethral duplication can also be associated with anorectal malformations, epispadias, hypospadias, bladder doubling, bladder exstrophy and other urinary excretory system anomalies [1,3,4].

## 2 Case report

**Fig. 1:**
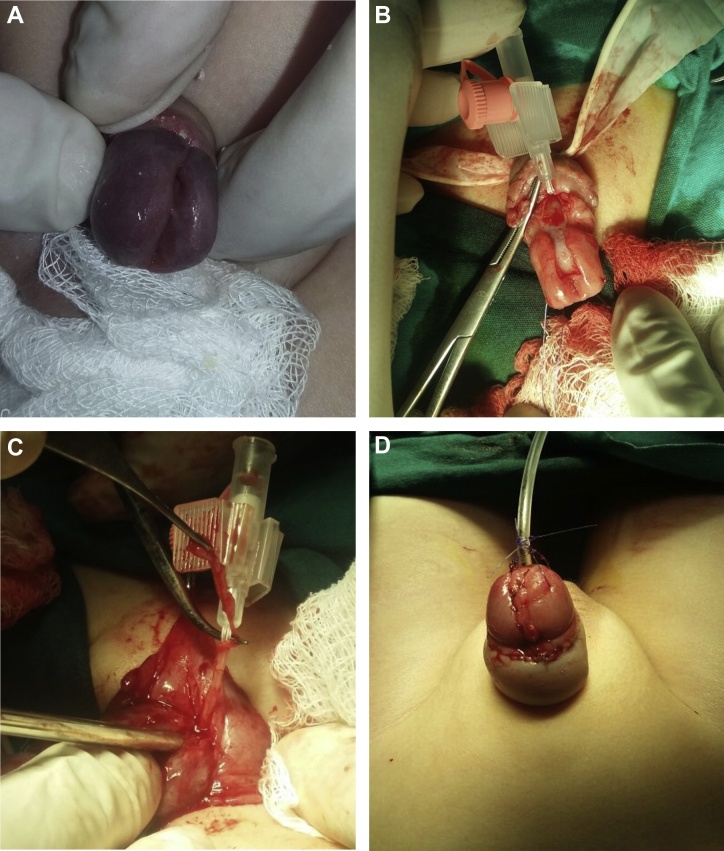
A-Urethral duplication on the dorsal surface of the genital organ; B, C-Removal of the accessory urethra; D-Condition of the penile after surgery.

A three-year-old boy was admitted to the Educational-Surgical clinicof Azerbaijan Medical University with diagnosis of “Duplication of urethra, Glanular epispadias” (Fig. 1A). There was done operations excision of the accessory urethra, urethroplasty and glanuloplasty on him. Patient with normaly urinary excretion, wasn't suffering from urinary incontinence and urinary tract infection. Parents of the patient have applied to the general surgeon for circumcision of their son. During the examination there was noted the splitting head of the genital organ and supernumerary urethra. Parents came to our clinic for the further examination and treatment of the patient.

In our examination there was detected the second - accessory urethra along with the main urethra on the dorsal surface of the penile. During the operation, the accessory urethra was stripped from the surrounding tissues to the root of the penile and it became apparent that the accessory urethra was merged into the main urethra at the same level (Fig. 1B, C). The accessory urethra was closed by suturing and removed from that part. Then, by using 7.0 pds thread there was performed urethroplasty with subcutaneous sutures on 10 Fr catheters on the dorsal surface of the penile in order to form the main urethra that has a defect. Subsequently, 10 Fr catheter was replaced with the 8 Fr catheter (Fig. 1D). Patient was discharged from hospital to home with an urinary catheter after 1 day. And after 1 week from surgery catheter was removed.

In the histological examination of the removed accessory urethra was not detected pathology. During the control examination urinary excretion and cosmetic appearance of the patient’s penile was significantly satisfactory.

## 3 Discussions

**Fig. 2:**
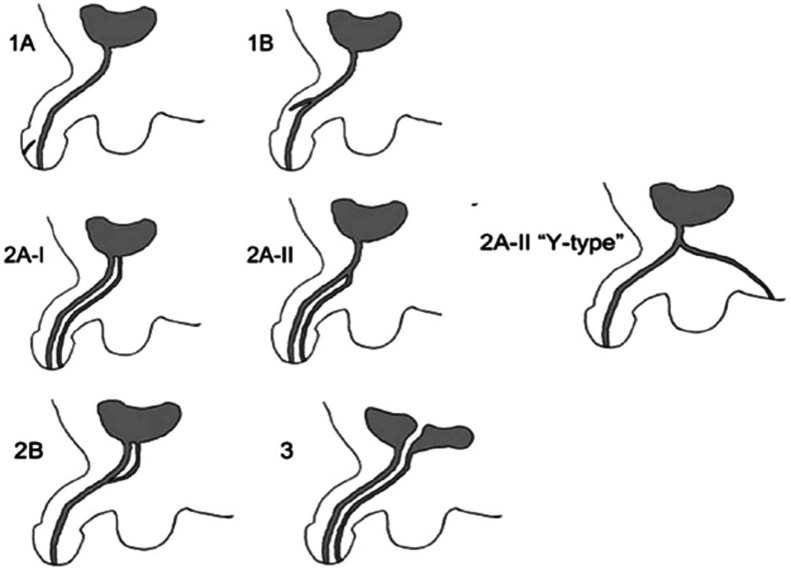
Effman classification: Type 1, blind incomplete urethral duplication; Type 1A (distal), opens on the distal or ventral surface of the penis but does not communicate with the urethra or bladder (the most common type); Type 1B (proximal), opens from urethral channel and ends blindly in the periurethral tissue (very rare type). Type 2, complete patent urethra duplication; Type 2A-I, two non-communicating urethras arising independently from the bladder or coursing independently to 2 different meatus; Type 2A-II, a second channel arising from the first and coursing independently to a second meatus (Y-type); Type 2B (one meatus), two urethras arising from the bladder or posterior urethra and uniting to form a common distal channel. Type 3, urethral duplication, which arises from duplicated or septated bladders.

Urethral duplication and epispadias are one of the rare anomalies of the genitourinary system. These anomalies can be observed both together and in isolated forms [5,6,8]. Despite the fact that there are many considerations about their embryology, the reason of appearance of these anomalies is still unknown [3]. Failure in the growth of Mullerian duct, ischemia, incorrect development of urogenital sinus are some of the accepted considerations [7,8]. In classification of urethral duplication on boys most often used a classification developed by Effman and his colleagues in 1976 (Fig. 2) [9]. According to this classification, along with the glanular epispadias in our patient was found a duplication similar to the type IIA-II of urethral duplication as well. Our patient’s main complaint was the splitting head of genital organ and incorrect location of the external urine hole. Ultrasound, voiding cystourethrography, retrograde urethrography, intravenous urethrography and cystoscopy are useful methods in making an exact diagnosis after patient’s clinical evaluation [2,10]. On the basis of the ultrasound, fluoroscopic, voiding cystourethrography examination for our patient we made diagnosis “Glanular epispadias and urethral duplication of type IIA-II”.

The treatment should be planned taking into account both the urinary system function and the cosmetic defect. Salle offered various surgical medicine approaches suitable to the type of urethral duplication [1]. Performing excision of the accessory urethra, urethroplasty and glanuloplasty on our patient we have formed penile with good cosmetic appearance.

## 4 Conclusions

Thereby, the mentioned clinical observation shows that although circumcision is a simple surgical procedure, but sometimes serious genital defects once again prove the importance of circumcision performed by pediatric surgeons and pediatric urologists. While planning of surgical treatment of such common anomalies along with the good cosmetic results the lower urinary excretory system functions should be considered as well.

## Author contribution

Study concept or design: Ramiz Polukhov, Vugar Mahammadov.

Data collection: Ramiz Polukhov, Vugar Mahammadov, Madina Baghirli.

Performed the analysis: Vugar Mahammadov, Madina Baghirli.

Writing the paper: Ramiz Polukhov, Madina Baghirli.

## Consent

Written informed consent was obtained from the patient’s parent for publication of this case report and accompanying images.

## Consent

Patient’s parents gave written consent for the publication of this case report.

## Ethical approval

Our study is exempt from ethical approval in Azerbaijan Medical University.

## Ethnical approval

Ethics Committee of Azerbaijan Medical University.

Figures: I approve that there no any details of author and patient.

## Research studies

It is case reports, not clinical trials or research.

## Funding

We have no source of funding.

## Guarantor

Ramiz Polukhov.

## Provenance and peer review

Not commissioned, externally peer reviewed.

## Declaration of Competing Interest

We have no any conflicts of interest.
